# Artemis (DCLRE1C) Acts as a Target to Enhance Radiotherapy Response in Triple-Negative Breast Cancer

**DOI:** 10.3390/cancers17203279

**Published:** 2025-10-10

**Authors:** Vasudeva Bhat, Kelsie L. Thu, Anayra de Fatima Goncalves Santiago, Anna C. Bonvissuto, Farhad Ghasemi, David Goodale, Michael V. Roes, Daniel T. Passos, Frederick A. Dick, David W. Cescon, Alison L. Allan, Armen Parsyan

**Affiliations:** 1 Department of Anatomy and Cell Biology, Schulich School of Medicine and Dentistry, Western University, London, ON N6A 3K7, Canada; vbhat@uwo.ca (V.B.); agonca6@uwo.ca (A.d.F.G.S.); apin@uwo.ca (A.C.B.); alison.allan@lhsc.on.ca (A.L.A.); 2 Verspeeten Family Cancer Centre, London Health Sciences Centre and London Health Sciences Centre Research Inc., London, ON N6A 5W9, Canada; david.goodale@lhsc.on.ca (D.G.); fdick@uwo.ca (F.A.D.); 3 Department of Oncology, Schulich School of Medicine and Dentistry, Western University, London, ON N6A 3K7, Canada; 4 Keenan Research Centre for Biomedical Science, St Michael’s Hospital Unity Health Toronto, Toronto, ON M5B 1T8, Canada; kelsie.thu@unityhealth.to; 5 Department of Laboratory Medicine and Pathobiology, University of Toronto, Toronto, ON M5S 1A8, Canada; 6 Division of General Surgery, Department of Surgery, Schulich School of Medicine and Dentistry, Western University, St Joseph’s Health Care and London Health Sciences Centre, London, ON N6A 4V2, Canada; 7 Department of Pathology and Laboratory Medicine, Schulich School of Medicine and Dentistry, Western University, London, ON N6A 5C1, Canada; mroes2028@meds.uwo.ca (M.V.R.); dpassos@uwo.ca (D.T.P.); 8 Department of Medical Oncology and Hematology, University of Toronto, Princess Margaret Cancer Centre, University Health Network, Toronto, ON M5G 2C1, Canada; dave.cescon@uhn.ca

**Keywords:** breast cancer, CRISPR screen, radiosensitizing target, DCLRE1C (Artemis), cellular senescence

## Abstract

Background/Objectives: The lack of canonical biomarkers and strategies to target radioresistance contribute to poor patient outcomes in triple-negative breast cancer (TNBC). Identifying and targeting novel radioresistance genes will benefit in enhancing radiotherapy response and treatment outcomes in TNBC patients. Methods: A genome-wide CRISPR screen was performed to identify radioresistance genes in the TNBC cell line. An in vitro clonogenic assay was used to assess the antiproliferative effects of Artemis knockout or pharmacologic inhibition of Artemis, either alone or in combination with RT. Tumor doubling time and animal survival were assessed using an in vivo xenograft model. RNA-seq analysis was performed to identify genes and pathways deregulated under Artemis knockout conditions, both alone and in combination with RT. Cellular senescence was evaluated using a β-galactosidase assay. Results: Our CRISPR screen identified Artemis as a top hit in RT-treated TNBC cells, whose depletion led to radiosensitization in TNBC. Artemis knockout significantly reduced cell proliferation and enhanced the antiproliferative effects of RT in vitro. Compared to mice-bearing control MDA-MB-231 xenografts, Artemis knockout exhibited prolonged survival that was further enhanced with RT. Bulk RNA-sequencing indicated that the antiproliferative and radiosensitization effects of Artemis depletion were mediated by the activation of cellular senescence which was confirmed with a β-galactosidase assay. Conclusions: Taken together, our results highlight the critical role of Artemis in TNBC cell proliferation and response to radiation. Our findings identify Artemis as a potential biomarker indicative of sensitivity to radiation and a putative target that could be inhibited to enhance the efficacy of RT in TNBC.

## Simple Summary

Patients with triple-negative breast cancer (TNBC) often experience poor patient outcomes due to a lack of reliable biomarkers and effective strategies to overcome radioresistance. In this study, we identify the novel roles of Artemis, both as a biomarker for radiosensitivity and a therapeutic target in TNBC. Using a genome-wide CRISPR-knockout screen, we identified Artemis as a key radiosensitizing target. Artemis knockout or pharmacological inhibition increased the sensitivity of TNBC cells to radiotherapy (RT), enhancing its anti-tumor effects. In animal models, Artemis knockout, in combination with RT, prolonged survival. Furthermore, RNA-seq analysis revealed that the activation of cellular senescence contributed to this enhanced therapeutic response in TNBC. Overall, our findings suggest that targeting Artemis could offer a new strategy to tailor and improve RT outcomes in TNBC patients.

## 1. Introduction

Breast cancer is the second leading cause of cancer-related deaths among women globally [1]. Triple-negative breast cancer (TNBC) is the most aggressive subtype of breast cancer. It is highly heterogeneous, lacks canonical biomarkers, and is often associated with treatment resistance, which leads to worse oncologic outcomes compared to other breast cancer subtypes [2,3]. Radiotherapy (RT) is a key modality for local and/or regional control at the primary tumor or metastatic disease sites in breast cancer [4,5,6]. However, resistance to RT is commonly observed in TNBC [7], leading to poor clinical outcomes [8,9,10]. Although the molecular mechanisms driving radioresistance in TNBC are not fully understood, recent studies point to DNA repair pathways [11], cell cycle redistribution [12], epithelial-to-mesenchymal transition (EMT) [13], and the tumor microenvironment [14] as potential contributors. Gaining deeper insights into these underlying molecular mechanisms may facilitate the identification of predictive biomarkers for RT response, paving the way for more effective and personalized treatment strategies for TNBC patients.

RT induces genotoxicity by generating DNA double-stranded breaks (DDSBs) [15], which are repaired primarily through homologous recombination (HR) or non-homologous end joining (NHEJ) DNA repair pathways [15]. Cancer cells exploit these repair mechanisms to evade genotoxic effects induced by RT, often leading to resistance [16]. Studies have demonstrated that targeting DNA repair pathway components is a promising strategy for sensitizing breast cancer cells to RT [17,18]. NHEJ is commonly activated in TNBC [19] and hence, disrupting this pathway could enhance TNBC cell sensitivity to RT. Several components of the NHEJ repair pathway such as the protein kinase DNA-PKcs, have been tested in pre-clinical models [20] but have shown increased toxicity and poor pharmacokinetics [21]. This suggests the need to identify novel targets that are specifically upregulated in TNBC tumors to enhance radiotherapy response.

In the current study, we employed a genome-wide CRISPR/Cas9 knockout screen to investigate the mechanisms of radioresistance in MDA-MB-231 TNBC cells. Our screen identified Artemis as a potential radiosensitizing target. Artemis is an endonuclease, encoded by *DNA Crosslink Repair 1C* (*DCLRE1C*), required for processing DNA ends, during V(D)J recombination [22] and NHEJ-mediated repair of RT-induced DDSBs [23]. Deficiency in Artemis has been linked to severe combined immunodeficiency with sensitivity to RT (RS-SCID) due to impaired V(D)J recombination [24,25]. Exon sequencing of breast cancer patients with the familial BRCA1/2 mutation identified nonsense mutations in the *DCLRE1C* gene [26], suggesting its role in breast cancer. However, the role of Artemis in breast cancer pathogenesis and therapy resistance, particularly in TNBC, is poorly understood.

Our findings demonstrated that the loss of function of Artemis led to a decrease in cell proliferation and enhanced anti-cancer effects of RT in TNBC cells both in vitro and in vivo. Furthermore, we found that targeting Artemis activity with non-oncology drugs such as ceftriaxone and auranofin, inhibitors of Artemis’ endonuclease activity in cell free assays [27], significantly enhanced RT response in TNBC cells. RNA-seq analysis revealed that a loss of Artemis resulted in the perturbation of genes related to cellular senescence that may potentially enhance RT. Taken together, our findings demonstrate that Artemis plays a significant role in TNBC proliferation and survival following RT. Therefore, these results indicate Artemis as a predictive biomarker and therapeutic target for enhancing radiosensitivity in TNBC.

## 2. Materials and Methods

### 2.1. Cell Culture Reagents

The MDA-MB-231 cell line was obtained from the American Type Culture Collection (ATCC, Manassas, VA, USA) and the SUM159 cell line was obtained from Asterand Inc. (Detroit, MI, USA). MDA-MB-231 cells were cultured in Dulbecco’s modified eagle medium (DMEM)/F12 supplemented with 10% fetal bovine serum (FBS). SUM159 cells were cultured in HAM/F-12 + 5% FBS, 1% HEPES, 0.5% insulin, and 0.1% hydrocortisone. MDA-MB-231 (ExPASy Callisaurus Research Resource Identifier RRID:CVCL_0062) and SUM159 (RRID: CVCL_5423) were authenticated via third-party testing on 04/2024 (IDEXX BioAnalytic, Columbia, MO, USA) using short tandem repeat profiling (using 9 markers) and were regularly tested to confirm mycoplasma negativity. The lenti-Cas9-blast transduced MDA-MB-231 cell line used for the CRISPR screen was obtained from the laboratory of Dr. DW Cescon [28]. The HEK-293T cells used for producing lentiviral particles were obtained from ATCC, Manassas, VA, USA.

### 2.2. Genome-Wide CRISPR/Cas9 Knockout Screening

Lentiviral particles encoding the genome-wide Moffat Toronto Knockout (TKO)-v2 pooled library [29] were generated by co-transfecting pooled TKO-v2 plasmids with psPAX2 (Addgene #12260) and pMD2.G (Addgene #12259) into HEK-293T cells using Lipofectamine^TM^ 2000 (Thermo Fisher Scientific, Waltham, MA, USA). MDA-MB-231-Cas9 cells were transduced with the TKO-v2 library at an MOI of ~0.3 using 8 µg/mL polybrene. The infection was performed on a low multiplicity of infections to ensure most of the cells were infected with a single sgRNA. Library transduced MDA-MB-23-Cas9 cells were selected with puromycin for 7 days (Supplementary Figure S1A) to generate a knockout cell pool. The CRISPR screening was performed in biological duplicates. The pools were expanded and treated with 0 Gy or 4 Gy radiation using the XRAD 225Cx (Precision) micro-IGRT delivery system. The cells were maintained for 25 days to obtain sufficient enrichment and depletion of sgRNAs. Genomic DNA (gDNA) was isolated from cells at day 0 and the surviving cells on Day 25 for both 0 Gy- and 4 Gy-treated conditions and subjected to sgRNA-targeted sequencing at the Princess Margaret Genome Centre (Toronto). Screens were performed with technical duplicates maintaining 200× library coverage. A relaxed cutoff of false discovery rate (FDR) of 25% was applied [30,31,32]. For quality control and normalization, EGFP and LacZ were used as control guides in addition to sgRNA targeting random loci on Chromosome 10 (Chr10Rand) and a low specificity target (Chr10Promiscous). sgRNA abundance between treatment conditions was statistically compared using the MAGeCK algorithm (v0.5.723) to identify screen hits, defined as genes targeted by sgRNA that were significantly depleted in the irradiated compared to non-irradiated condition.

### 2.3. CRISPR/Cas9 Artemis-Specific Knockout

MDA-MB-231 and SUM159 cells were used to generate the Artemis knockout lines. Four different sgRNA sequences (Supplementary Table S1A) targeting Artemis (*DCLRE1C*) were selected from the TKO-v2 sgRNA library, and a scrambled sgRNA was used as a control. For each sgRNA, oligos were annealed and individually inserted into the LentiCRISPRv2 lentiviral vector (Addgene, Watertown MA, USA, #52691). Lentivirus was made as described. Viral particles (sgControl, sgDCLRE1C #1, #2, #3, and #4) were transduced into MDA-MB-231 and SUM159 cells using 8 µg/mL polybrene. After 24 h, cells were selected using 2 µg/mL (MDA-MB-231) and 2.5 µg/mL (SUM159) puromycin for 48 h. All the knockout lines were confirmed to be negative for mycoplasma.

### 2.4. The Cancer Genome Atlas (TCGA) Data Analysis

Clinical data from TCGA was analyzed using the University of ALabama at Birmingham CANcer (UALCAN) (https://ualcan.path.uab.edu/index.html) interactive web resource [33,34]. The expression of genes in breast cancer patients was analyzed using the TCGA breast invasive carcinoma (BRCA) data set. Survival analysis of TNBC patients with high and low *DCLRE1C* expression through media was performed using the Gene Expression Profiling Interactive Analysis (GEPIA2) web-based tool (http://gepia2.cancer-pku.cn/#survival (accessed on 20 December 2024)). The BRCA data set, with a basal-like/triple-negative subtype filter, was used to generate a survival plot.

### 2.5. Colony-Forming Assay

Colony formation assays (CFAs) were performed as previously described [35,36,37]. Parental and CRISPR-knockout MDA-MB-231 and parental SUM159 TNBC cells were plated singly at a density of ~20 cells/cm^2^ of 9.6 cm^2^ cell culture dish. The following day (16–20 h after seeding), the cells were exposed to an ID50 dose of radiation (2 Gy for MDA-MB-231 and 5 Gy for SUM159) [35,38] using a Cobalt-60 irradiator at the Verspeeten Family Cancer Centre (London, ON, Canada). Immediately after irradiation, cells were replenished with media containing either DMSO (0.1%; equivalent to the highest dose of the drug) or varying concentrations (0.01 µM, 0.1 µM, 1 µM, 10 µM, and 100 µM) of ceftriaxone (Cat. No. C5793, Millipore Sigma, Darmstadt, Germany), auranofin (Cat. No. A6733, Millipore Sigma, Darmstadt, Germany) and ampicillin (Cat. No. BP1760-5, Thermo Fisher Scientific Inc., Pittsburg, PA, USA).

### 2.6. In Vivo Studies

Animal experiments were carried out in accordance with the Canadian Council for Animal Care under a protocol approved by the University of Western Ontario Animal Care Committee (#2022-43). MDA-MB-231 sgControl and sgDCLRE1C_#2 lines were exposed to either 2 Gy or no radiation (0 Gy). Subsequently, the cells were trypsinized and viability was assessed using trypan blue. Cells were then resuspended in Hank’s Balanced Salt Solution (HBSS) at a concentration of 1 × 10^7^ viable cells/mL. Cell suspensions (100 µL; 1 × 10^6^ cells/mouse) were injected into the mammary fat pad (m.f.p) of 6–8-week-old female NOD/SCID mice (*n* = 5 mice/group). The size of the primary tumor was assessed weekly for 6 months using a digital caliper measurement in 2 perpendicular dimensions and tumor volume was calculated using the formula: volume = 0.52 × (width)^2^ × (length). Mice were sacrificed once the tumor volume reached 1500 mm^3^.

### 2.7. Immunoblotting

MDA-MB-231 sgControl, MDA-MB-231 sgDCLRE1C (#1–#4), SUM159 sgControl, and SUM159 sgDCLRE1C (#1, #3, and #4) cell lines were lysed using 1X RIPA lysis buffer (Cat. No. 89900, Thermo Scientific, Austin, TX, USA) containing 1X protease inhibitor (Cat. No. 78430, Thermo Scientific) and 1X phosphatase inhibitor (Cat. No. 1862495, Thermo Scientific). Protein (30 µg per sample; quantified by the Lowry assay) was boiled for 5 min in 1X gel loading buffer. The samples were then subjected to sodium dodecyl-sulfate polyacrylamide gel electrophoresis (SDS-PAGE) and transferred onto a polyvinylidene fluoride membrane (PVDF, Cat. No. 88518, Thermo Scientific). Membranes were blocked using either 5% bovine serum albumin (BSA) or 5% skimmed milk powder in Tris-buffer saline + 0.1% Tween-20 (TBST) for 1 h at room temperature prior to incubation with the primary antibody (Supplementary Table S1B) diluted in 5% blocking solution for overnight at 4° Celsius. Blots were then incubated with secondary antibody (Supplementary Table S1B) conjugated to horse radish peroxidase (HRP) for 1 h at room temperature. The Amersham ECL Primer Detection Reagent (GE Healthcare, Milwaukee, WI, USA) was used to measure protein expression by visualizing chemiluminescence using the BioRad ChemiDoc^TM^ MP imaging system. The blot was stripped using a stripping buffer (1.5% glycine, 0.1% SDS, 1% tween-20) and incubated with anti-Actin-HRP conjugated antibody (1:25,000, Cat. No. ab49900, Abcam, Cambridge, MA, USA) for 1 h at room temperature and protein expression was measured as described above. The densitometric analysis was performed using Image Lab software (v6.1, Bio-Rad Laboratories, Inc., Hercules, CA, USA).

### 2.8. Cell Cycle Analysis

The sgControl, sgDCLRE1C#2 MDA-MB-231, sgControl, and sgDCLRE1C#3 SUM159 cells were plated at ~7250 cells/cm^2^ in a 35 cm^2^ cell culture dish. The following day (16–20 h after seeding), control (sgControl), Artemis knockout (sgDCLRE1C) MDA-MB-231, and SUM159 cells were exposed to either a 0 Gy or 2 Gy (MDA-MB-231) dose of RT and either 0 Gy or 5 Gy dose of RT (SUM159). The media was replenished immediately after irradiation and cells were cultured for 24 h. Cells were trypsinized, washed, and centrifuged at 1000× *g* for 5 min. The pellet was resuspended in 0.4 mL of 1X PBS and 1 mL of pre-chilled 100% ethanol was added dropwise to the cell suspension with gentle vortexing. Cells were then centrifuged at 200× *g* for 3 min at 4 °C and washed twice with 1X PBS. The cell pellet was resuspended in PBS containing 100 µg/mL RNase A and 50 µg/mL propidium iodide and incubated at room temperature for 30 min. Cell cycle data were acquired using were then analyzed using a Beckman Coulter FC500 flow cytometer. A total of 10,000 events were captured. The data were analyzed using FlowJo software (v10.6.2).

### 2.9. Galactosidase Staining

Control and Artemis knockout MDA-MB-231 and SUM159 cells were seeded at a density of ~5000 cells per/cm^2^ of a 9.6 cm^2^ cell culture dish. The following day (16–20 h after seeding), the cells were exposed to either a 0 Gy or 2 Gy dose of RT. Immediately after irradiation, cells were replenished with media and incubated for 72 h. The senescence β-galactosidase staining kit (C, Cat. No. 9860, Cell Signaling Technology, Beverly, MA, USA) was used to characterize senescent cells according to the manufacturer’s protocol. The stained cells were imaged using an Olympus CKX53 microscope and β-galactosidase positive cells were counted using ImageJ software (Bethesda, MD, USA).

### 2.10. RNA-Seq Analysis

MDA-MB-231 sgControl and sgDCLRE1C#2 cell lines were exposed to either 0 Gy or 2 Gy radiation (previously determined ID50 dose) [35,38]. Immediately after irradiation, the growth medium was refreshed. Total RNA was harvested 72 h post-irradiation using TRIzol^®^ (Cat. No. 15596-026, Thermo Scientific) reagent as per the manufacturer’s instruction and quantity and quality measurements were performed using NanoDrop One (Thermo Scientific). Samples were analyzed in the London Regional Genomics Centre (LRGC) for quality assessment using an Agilent 2100 Bioanalyzer, rRNA reduction, and creation of indexed libraries. RNA sequencing was performed using an Illumina NextSeq High 75 cycle Sequencing Kit and RNA-seq analysis was performed using R software. Raw counts were imported into the R statistical environment (version 4.4.1). The DESeq2 (version 1.44) [39] package was used for differential gene expression analysis. Independent filtering to remove genes with low counts was carried out as per the DESeq2 default protocol. The Benjamin–Hochberg method was used to adjust the *p* value for multiple testing, and a threshold of 0.05 was used for FDR. A PCA plot was generated using the 500 genes with the greatest variance in expression as input after normalizing the expression counts through variance stabilizing transformation (VST). Volcano plots were generated using ggplot2 (v.3.5.1) and ggrepel (v.0.9.5) packages. Differentially expressed genes (DEGs) were visualized in a volcano plot using the EnhancedVolcano package (https://github.com/kevinblighe/EnhancedVolcano; accessed on 24 October 2024). The dplyr and ggplot2 packages were used to generate graphs. Time-to-event was analyzed using Kaplan–Meier curves and the log-rank test to compare sgControl_2Gy, sgDCLRE1C_0Gy, and sgDCLRE1C_2Gy to sgControl_0Gy. Hazard ratios with 95% confidence intervals were estimated using Cox’s proportional hazard model. Analyses were conducted using R with the survival and survminer packages.

### 2.11. Pathway Analysis and Gene Set Enrichment Analysis (GSEA)

Gene set enrichment analysis (GSEA) was performed using the GSEA Preranked tool (version 4.3.3) [40,41] to identify enriched gene sets under different treatment conditions. Genes were ranked based on the numeric ranking statistics. The enriched pathways with FDR q-value < 0.05 were considered significant. Pathway analysis was conducted using the Reactome package [42] to identify pathways enrichment for DEGs under different treatment conditions.

### 2.12. Reverse Transcription Quantitative PCR (RT-qPCR)

Control and Artemis knockout MDA-MB-231 and SUM159 cells were exposed to either a 0 Gy or 2 Gy and 0 Gy or 5 Gy dose of RT, respectively. After 72 h, total RNA was extracted using TRIzol^TM^ reagent (Invitrogen, Carlsbad, CA, USA). An amount of 1 µg of RNA was used to synthesize cDNA using Superscript IV VILO Master Mix (Invitrogen). Gene expression changes under different conditions were assessed by reverse transcription quantitative PCR using Brilliant III SYBR green qPCR master mix (Agilent Technologies, Inc.). All reactions were carried out in triplicate for each biological replicate and the fold change in different groups vs. control was calculated using the 2^−ΔΔCt^ method as previously described [43]. GAPDH was used for normalization.

### 2.13. Statistical Analysis

All in vitro experiments were performed in triplicate (*n* = 3) unless stated otherwise. in vivo experiments were carried out using 5 mice per group (total *n* = 20 mice). All statistical analyses were carried out using GraphPad Prism 6.0 (San Diego, CA, USA). All data are represented as the mean ± standard error of mean (SEM). A two-way analysis of variance (ANOVA) was used to compare multiple means across different groups. In all cases, *p*-values ≤ 0.05 were considered to be statistically significant.

## 3. Results

### 3.1. Genome-Wide CRISPR Knockout Screen Identifies ARTEMIS as the Top Radiosensitizing Target

**Figure 1:**
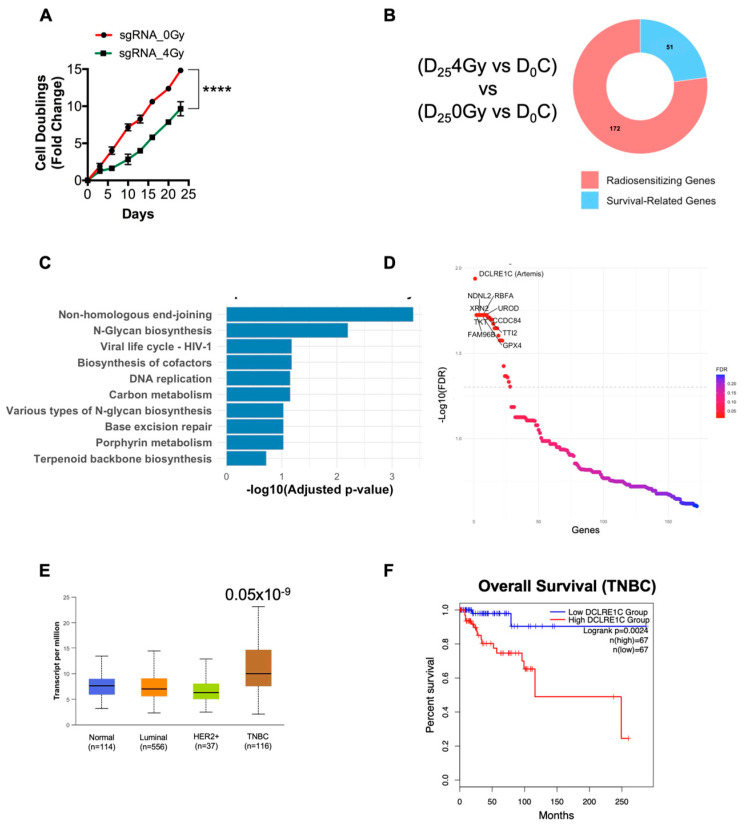
**CRISPR screen identifies Artemis (DCLRE1C) as a candidate for radiosensitizing targets in TNBC.** (**A**) Graph shows the cell doubling time of CRISPR knockout MDA-MB-231 cell lines exposed to either 0 or 4 Gy of RT. Cell doubling was calculated by counting cells at every cell passage for 25 days post-RT. **** = *p* ≤ 0.0001. (**B**) Donut chart shows the number of potential radiosensitizing targets and survival-related genes identified in the CRISPR knockout screen in TNBC cells exposed to RT. (**C**) KEGG pathway analysis was performed to identify significantly overrepresented pathways. (**D**) Sigmoid plot shows the list of top hit genes identified in the CRISPR screen, whose depletion may sensitize TNBC cells to RT. (**E**) Using the UALCAN interactive web resource, TCGA-BRCA data were analyzed to examine the expression of the NHEJ pathway genes in breast cancer. Expression of *DCLRE1C* in TNBC patient samples as compared to normal breast tissue samples (*p* ≤ 0.05 × 10^−9^). (**F**) The GEPIA2 web-based tool was used to perform survival analysis on TNBC patients with high and low *DCLRE1C* (Artemis) expression. A lower expression of *DCLRE1C* was associated with better overall survival when compared to patients with high *DCLRE1C* expression.

To identify potential genes involved in radioresistance, we performed a genome-wide CRISPR-Cas9 dropout screen in MDA-MB-231 TNBC cells. MDA-MB-231 cells expressing Cas9 were transduced with the human Moffat TKO-v2 library which contains 90,000 unique single guide RNAs (sgRNAs), targeting approximately 18,053 protein-coding genes with 3–5 unique sgRNAs per gene. Targeted sgRNA sequencing on genomic DNA (gDNA) from a fraction of cells collected pre-treatment was performed to determine baseline sgRNA abundance (day 0, D_0_). The remaining cells were treated with 0 Gy (non-irradiated control) and 4 Gy of radiation and subsequently passaged and cultured for 25 days (D_25_), at which point gDNA was collected for sgRNA sequencing (Supplementary Figure S1A). We found that 4 Gy RT significantly increased the doubling time of TKO-v2 library infected cells compared to the no-RT control (Figure 1A). We then analyzed the sgRNA-sequencing data to identify sgRNAs that were selectively depleted in the D_25_4Gy-treated cells compared to D_0_C (Figure 1B, Supplementary Figure S1A). These depleted sgRNAs have a high probability of representing genes whose loss-of-function sensitized MDA-MB-231 cells to RT. The putative radiosensitizing target from D_25_4Gy vs. D_0_C were then cross referenced to the D_25_C vs. D_0_ gene list to identify hits specific to RT and to exclude survival-related genes. This identified 172 potential radiosensitizing targets (Figure 1B, Supplementary Table S2). Kyoto Encyclopedia of Genes and Genomes (KEGG) pathway analysis on the candidate gene list revealed that pathways such as NHEJ, DNA replication, and base excision repair were significantly overrepresented (Figure 1C) suggesting that the inactivation of the components of these pathways sensitized MDA-MB-231 cells to RT.

We then focused on the top 10 radiosensitizing targets identified from our screen. We found that *DCLRE1C* was the top hit in our screen, representing a potential gene whose loss of function resulted in the radiosensitization of TNBC cells (Figure 1D). We also identified additional genes, including families with sequence similarity 96 member B (FAM96B), Transketolase (TKT), Necdin-like 2 (NDNL2), 5′-3′ Exoribonuclease 2 (XRN2), Glutathione peroxidase 4 (GPX4), Ribosome binding factor A (RBFA), TELO2 interacting protein 2 (TTI2), Uroporphyrinogen decarboxylase (UROD), and Coiled-coil domain-containing 84 (CCDC84) as the top 10 hits in our screen (Figure 1D). Evidence suggested that most of these genes were directly or indirectly involved in conferring resistance to RT and chemotherapies [44,45,46,47,48,49,50,51].

TCGA analysis revealed that *DCLRE1C* (Artemis) mRNA expression was significantly upregulated in the TNBC subtype when compared to other breast cancer subtypes and normal tissue (Figure 1E). Furthermore, survival analysis using the GEPIA2 web-based tool revealed that a higher expression of *DCLRE1C* correlates with poor overall survival of TNBC patients as compared to patients with low *DCLRE1C* levels (Figure 1F).

### 3.2. Loss of Function of Artemis Significantly Decreases Proliferation and Enhances RT Response in TNBC Cells In Vitro

**Figure 2:**
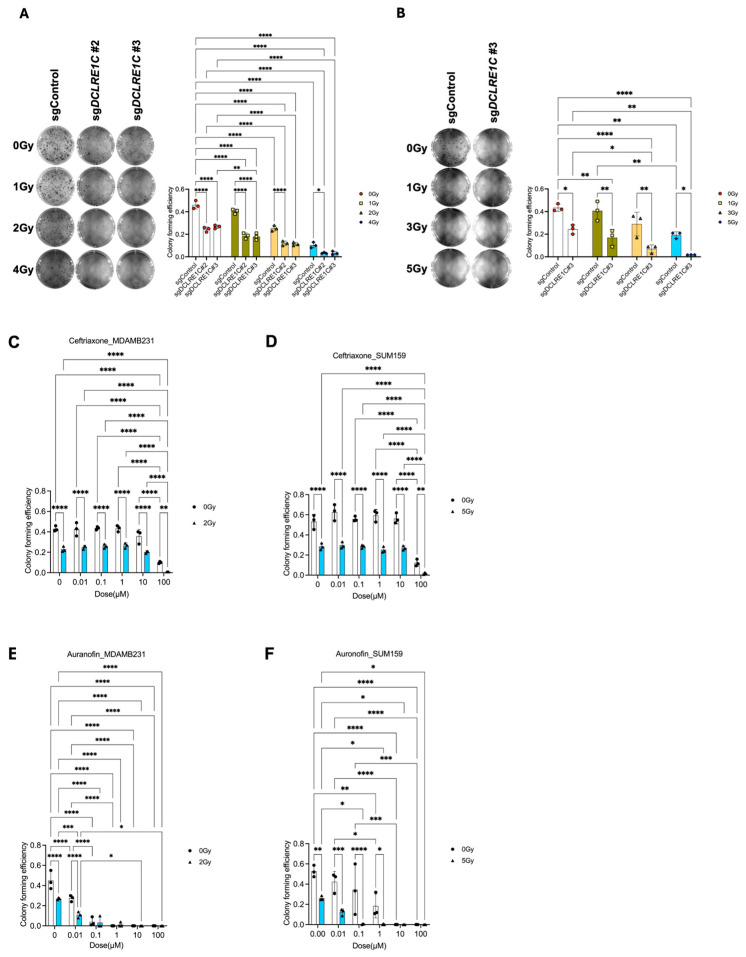
Loss of function or pharmacological targeting of Artemis leads to anti-proliferative effects and radiosensitization in TNBC. (**A**) Left: Representative image showing the colonies formed in sgControl and sgDCLRE1C#2 and sgDCLRE1C#3 MDA-MB-231 cell lines exposed to different doses of radiation (0, 1, 2 and 4 Gy). Right: The bar graph shows the colony-forming efficiency of sgControl, sgDCLRE1C#2, and sgDCLRE1C #3 MDA-MB-231 cell lines exposed to different doses of RT. (**B**) Left: Representative image showing the colonies formed in sgControl and sgDCLRE1C#3 SUM159 cell lines exposed to different doses of radiation (0, 1, 3, and 5 Gy). Right: The bar graph shows the colony-forming efficiency of sgControl and sgDCLRE1C#3 SUM159 cell lines exposed to different doses of radiation. * = *p* ≤ 0.05, ** = *p* ≤ 0.01, and **** = *p* ≤ 0.0001. (**C**–**F**) MDA-MB-231 and SUM159 TNBC cells were grown in colony-forming assay conditions and treated with different doses of the drug in the absence or presence of ID50 dose (2 Gy for MDA-MB-231, 5 Gy for SUM159) of RT. Ceftriaxone (B&C) and auranofin (D&E) showed a dose-dependent decrease in colony forming ability and enhanced effects RT in TNBC cells. * = <0.05, ** = 0.01, *** = 0.001, and **** = 0.0001.

To further investigate the role of Artemis in TNBC, we generated Artemis CRISPR-knockout lines using four unique sgRNAs targeting *DCLRE1C* gene (sgDCLRE1C#1–#4). A non-targeting sgRNA (sgControl) was used to generate a control cell line wildtype for Artemis. Knockout lines that showed complete depletion of *DCLRE1C* (Supplementary Figure S1B,C) in immunoblotting analysis were used for functional analysis. To investigate the role of Artemis in regulating RT response, we performed CFA using control and CRISPR-knockout cell lines. The control and Artemis knockout MDA-MB-231 cells were exposed to different doses of RT (0, 1, 2 and 4 Gy), and after 7 days, the difference in colony-forming efficiency was calculated. We observed a dose-dependent decrease (1.14, 1.8, and 4.6-fold) in colony numbers in MDA-MB-231 sgControl cells when compared to 0 Gy (Figure 2A). We found a further reduction in colony numbers in MDA-MB-231 sgDCLRE1C#2; 1.9-fold (0 Gy), 2.5-fold (1 Gy), 4-fold (2 Gy), 14-fold (4 Gy), and #3; 1.7-fold (0 Gy), 2.6-fold (1 Gy), 4-fold (2 Gy), and 13.9-fold (4 Gy) in response to RT, compared to 0 Gy and sgControl (Figure 2A).

Control and Artemis knockout SUM159 cells exposed to different doses of RT (0, 1, 3, and 5 Gy) demonstrated similar results. We observed a dose-dependent decrease (1.1, 1.5, and 2.2-fold) in colony numbers in SUM159 sgControl cells as compared to 0 Gy (Figure 2B). This reduction in colony-forming ability was further enhanced in SUM159 sgDCLRE1C#3; 1.7-fold (0 Gy), 2.4-fold (1 Gy), 4.1-fold (3 Gy), and 10.7-fold (5 Gy) in response to different RT doses (Figure 2B). These findings suggest that a loss of Artemis function leads to a decrease in clonogenic survival, validating Artemis as a top hit in our screen.

### 3.3. Putative Pharmacological Inhibitors of Artemis Exert Antiproliferative and Radiosensitization Effects in TNBC

To investigated the possibility of targeting Artemis to enhance radiosensitivity in the clinic, we utilized non-oncology drugs previously reported to inhibit Artemis activity [27,52] in cell-free assays [53], including ceftriaxone, ampicillin, and auranofin. We found that 100 µM of ceftriaxone resulted in a 16- and 4-fold (*p*-value < 0.0001) decrease in colony numbers in parental MDA-MB-231 and SUM159 cells, respectively (Figure 2C,D). Notably, this decrease was further enhanced (43- and 30-fold; *p*-value < 0.0001) in the presence of RT (ID50 dose of RT [35,38]) in both cell lines (Figure 2C,D) as compared to the control.

Auranofin treatment at 0.1 µM concentration induced an 11-fold and 1.5-fold (*p*-value < 0.0001) decrease in colony formation in parental MDA-MB-231 (Figure 2E) and SUM159 (Figure 2F) cells, respectively, compared to vehicle-treated control cells. This decrease in colony numbers was further enhanced by the presence of RT in both cell lines as compared to the control (13-fold in MDA-MB-231 and 94-fold in SUM159) (Figure 2E,F). However, ampicillin did not have a significant effect on colony-forming efficiency in TNBC cells (Supplementary Figure S1D,E). These results suggested that the pharmacological inhibition of Artemis exerted anti-proliferative effects on TNBC cells, which are further enhanced in the presence of RT.

### 3.4. Loss of Function of Artemis Significantly Impairs Growth and Enhances Radiotherapy Response of TNBC Tumors In Vivo

**Figure 3:**
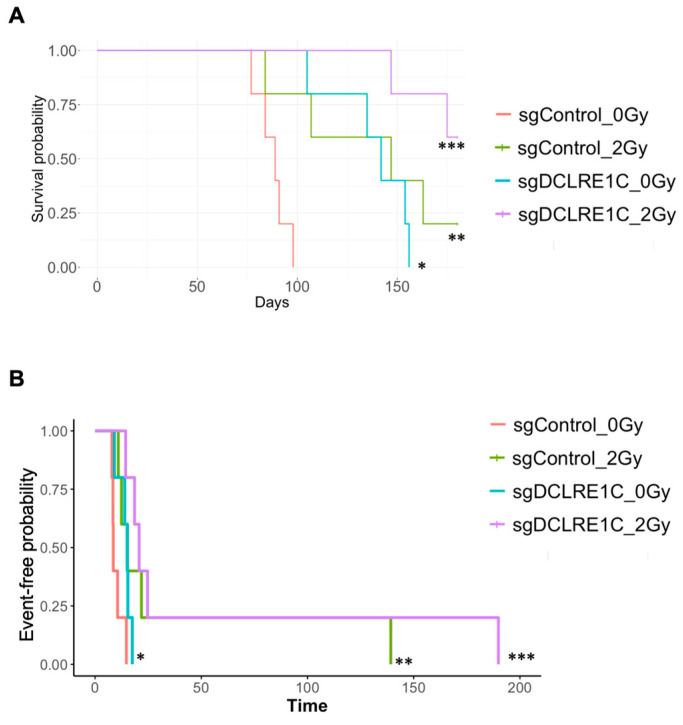
**Loss of Artemis in combination with RT prolonged survival of mouse xenografts.** The sgControl and sgDCLRE1C#2 MDA-MB-231 cells were exposed to either no radiation (0 Gy) or 2 Gy RT and injected (viable cells) into the mammary fat pads of 6–8-week-old female NOD/SCID mice. (**A**) Kaplan–Maier survival curves show the time taken for the mice injected with sgControl_0Gy, sgControl_2Gy, sgDCLRE1C#2_0Gy, and sgDCLRE1C #2_2Gy to reach the humane endpoint. * = *p* ≤ 0.01, ** = *p* ≤ 0.005, and *** = *p* ≤ 0.001. (**B**) Kaplan–Meier time to doubling shows the time taken for the tumor to double in size in the mice injected with sgControl_2Gy (hazard ratio: 0.10; 95% CI: 0.02–0.49; *p* value = 0.005), sgDCLRE1C#2_0Gy (hazard ratio: 0.25; 95% CI: 0.06–1.00; *p* value = 0.049), or sgDCLRE1C#2_2Gy (hazard ratio: 0.06; 95% CI: 0.01–0.30; *p* value = <0.001) MDA-MB-231 cells compared to sgControl_0Gy.

To assess the role of Artemis in vivo, both sgControl and sgDCLRE1C#2 MDA-MB-231 cells were exposed to either 0 Gy or 2 Gy (ID50 dose) radiation, and 1 × 10^6^ viable cells were injected into 4–6 weeks old female NOD-SCID mice (Supplementary Figure S1F). Compared to mice in the sgControl_0Gy group (median 89 days), the median time to reach the humane endpoint (1500 mm^3^ tumor volume) was significantly extended in the sgControl_2Gy (median 147 days; *p* < 0.005), sgDCLRE1C_0Gy (median 142 days; <0.01), and sgDCLRE1C_2Gy (median > 165 days; *p* < 0.001) (Figure 3A) groups. Additionally, we found that the doubling time of the tumor in sgControl_2Gy, sgDCLRE1C_0Gy, and sgDCLRE1C_2Gy conditions were significantly greater compared to sgControl_0Gy (Figure 3B). Taken together, our data demonstrate that the loss of function of Artemis alone or in combination with RT significantly reduces TNBC growth and improves survival of tumor-bearing mice.

### 3.5. Loss of Artemis Expression Activates Anti-Cancer Phenotypes in TNBC

**Figure 4:**
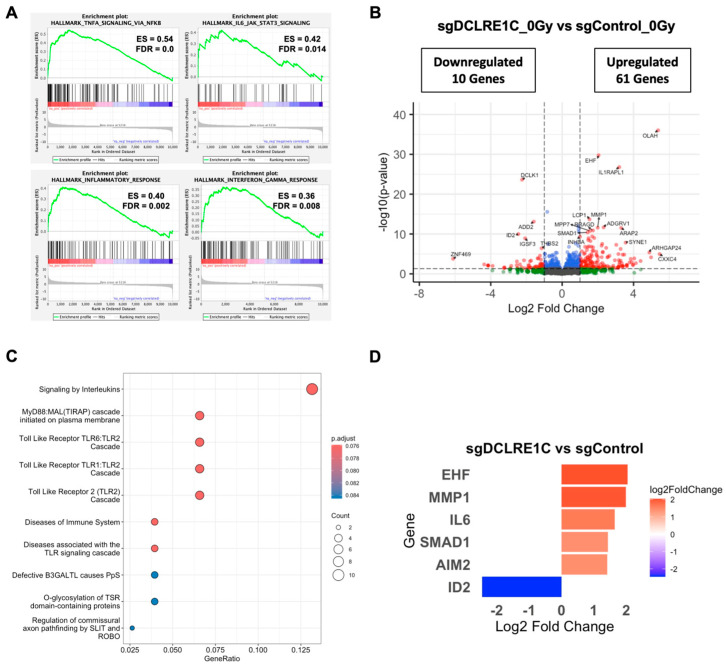
**RNA-seq analysis identifies differentially expressed genes (DEGs) and enriched pathways upon Artemis depletion.** (**A**) Gene set enrichment analysis (GSEA) plot shows positively enriched gene sets upon Artemis depletion relative to control. (**B**) Volcano plot shows DEGs in Artemis-deficient cells compared to control. Red dots represent significantly up- and downregulated genes (fold change ≥1.5 and FDR ≤ 0.05), while blue dots represent significantly changed genes (FDR <0.05) with fold change <1.5. (**C**) Dot plot shows significantly overrepresented pathways in MDA-MB-231 cells lacking Artemis as compared to control. (**D**) Bar graph shows the expression of pro- and anti-tumorigenic genes in Artemis-deficient cells as compared to control.

To explore the mechanisms underlying the observed antiproliferative and radiosensitization effects mediated by the loss of Artemis, we performed bulk RNA-seq on control and Artemis knockout cells exposed to either 0 or 2 Gy of RT. Whole transcriptomic profiles of the biological replicates clustered separately for each treatment condition (Supplementary Figure S2A). Gene set enrichment analysis (GSEA) of sgDCLRE1C_0Gy vs. sgControl_0Gy for hallmark pathways revealed significant (FDR-q-value < 0.05) enrichment of various inflammatory- and senescence-associated [54,55,56,57,58,59] gene sets including tumor necrosis factor alpha (TNFA) signaling via NFκB, IL6/JAK/STAT3 signaling, inflammatory response, and interferon gamma response (Figure 4A, Supplementary Table S3). We then restricted our analysis to a most significantly perturbed gene list (adjusted *p*-value < 0.05). Compared to sgControl, we observed 122 differentially expressed genes (DEGs) in Artemis knockout cells (Supplementary Table S4). Among these DEGs, 61 genes were significantly upregulated (log2 foldchange ≥ 1.0), and 10 were significantly downregulated (log2 foldchange ≤ −1.0) (Figure 4B, Supplementary Table S4). Furthermore, the Reactome pathway analysis on these genes revealed the enrichment of inflammatory pathways such as Toll-like receptors and interleukin signaling (Figure 4C). We found a number of DEGs associated with cellular senescence and SASP that were upregulated including the following: absent in melanoma 2 (AIM2) [60], ETS homologous factor (EHF) [61], interleukin 6 (IL6) [62], matrix metallopeptidase 1 (MMP1) [63], Toll-like receptor 4 (TLR4) [59], and SMAD family member 1 (SMAD1) [64]; Inhibitor of DNA binding 2 (ID2) [65], which is involved in the inhibition of senescence, was significantly downregulated in the absence of Artemis (Figure 4D).

To further understand the clinical relevance of DEGs upon Artemis depletion, we analyzed gene expression patterns across different breast cancer subtypes using the TCGA-clinical invasive carcinoma database. We found that most of the SASP-associated genes upregulated in the absence of Artemis had a significantly lower gene expression in TNBC samples compared to normal breast tissue (Supplementary Figure S2B). Similarly, genes downregulated in the Artemis knockout condition had higher expression in TNBC samples compared to normal tissue samples (Supplementary Figure S2B). This difference was further validated by RT-qPCR in the control and Artemis knockout MDA-MB-231 and SUM159 cells exposed to either no radiation or their respective ID50 dose of radiation (Supplementary Figure S2C,D).

### 3.6. Artemis Deficiency Further Enhances RT Effects in TNBC via Induction of G2/M Arrest and Senescence

**Figure 5:**
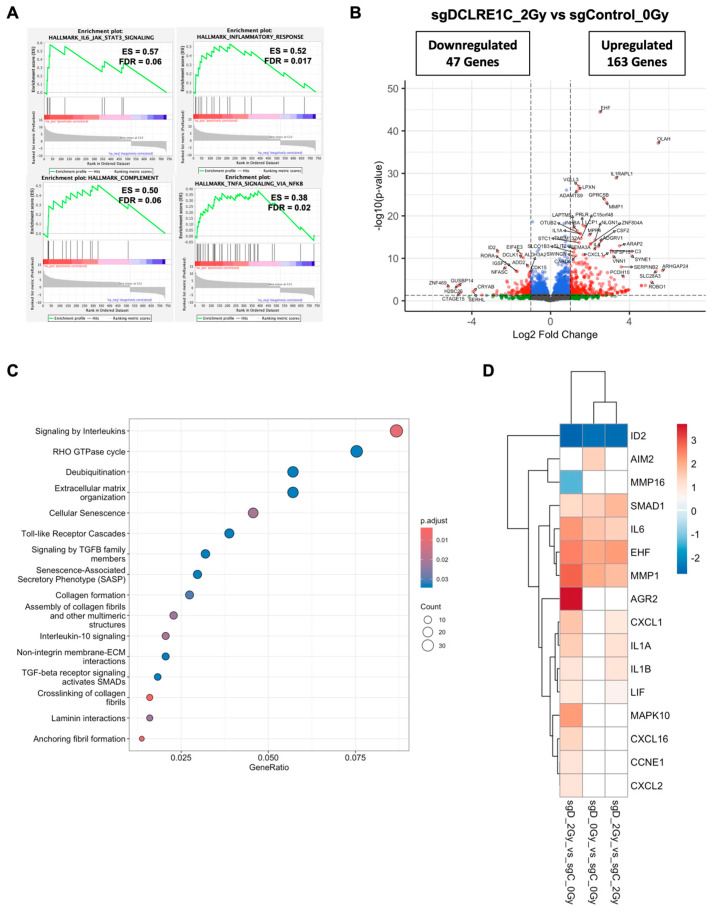
**Artemis depletion enhances RT response via the activation of cellular senescence related genes.** (**A**) GSEA reveals positive enrichment of IL6/JAK/STAT3, inflammatory response, TNFA/NFκB, and complement pathway gene sets in radiation treated, Artemis knockout cells relative to non-irradiated control. (**B**) Volcano plot shows the differentially expressed genes (DEGs) in radiation treated, Artemis-depleted cells compared to non-irradiated control. Red dots represent significantly up- and downregulated genes (fold change ≥1.5 and FDR ≤ 0.05), while blue dots represent significantly changed genes (FDR <0.05) with fold change <1.5. (**C**) Dot plot shows overrepresented signaling pathways under Artemis knockout conditions combined with RT. (**D**) Heatmap depicting perturbation of genes related to cellular senescence in sgDCLRE1C_0Gy vs. sgControl_0Gy, sgDCLRE1C_2Gy vs. sgControl_0Gy, and sgDCLRE1C_2Gy vs. sgControl_2Gy. Shades of red indicate significantly upregulated genes, blue indicates significantly downregulated genes, and the white represents non-significant changes.

To investigate how the loss of Artemis enhanced RT response, we analyzed DEGs in sgDCLRE1C_2Gy as compared to sgControl_0Gy. Similar to the Artemis knockout condition alone, GSEA revealed an enrichment of IL6/JAK/STAT3 signaling, inflammatory response, and TNFA/NFκB signaling gene sets (Figure 5A) in Artemis knockout cells exposed to RT relative to control cells (sgControl_0Gy). RT, under Artemis knockout conditions, resulted in the perturbation of 734 genes, with 163 upregulated and 47 downregulated genes (Figure 5B). Furthermore, the Reactome pathway analysis revealed the overrepresentation of pathways associated with cellular senescence, SASP, interleukin, and Toll-like receptor signaling in radiation-treated TNBC cells lacking Artemis expression (Figure 5C). Consistent with this result, we found the perturbation of additional senescence/SASP-associated genes in the presence of RT (Figure 5D). We also analyzed DEGs in sgDCLRE1C_2Gy compared to sgControl_2Gy. Although the enrichment of IL6/JAK/STAT3 signaling (Supplementary Table S3) was not statistically significant in our GSEA, we observed perturbation of senescence/SASP-associated genes (Figure 5D). When DEGs from non-irradiated Artemis knockout cells (Figure 4B) were compared to DEGs from radiation-treated Artemis knockout cells (Figure 5B), we found pathways related to extracellular matrix organization, cellular senescence, SASPs, and DNA-damage to be significantly enriched (Supplementary Figure S3A).

**Figure 6:**
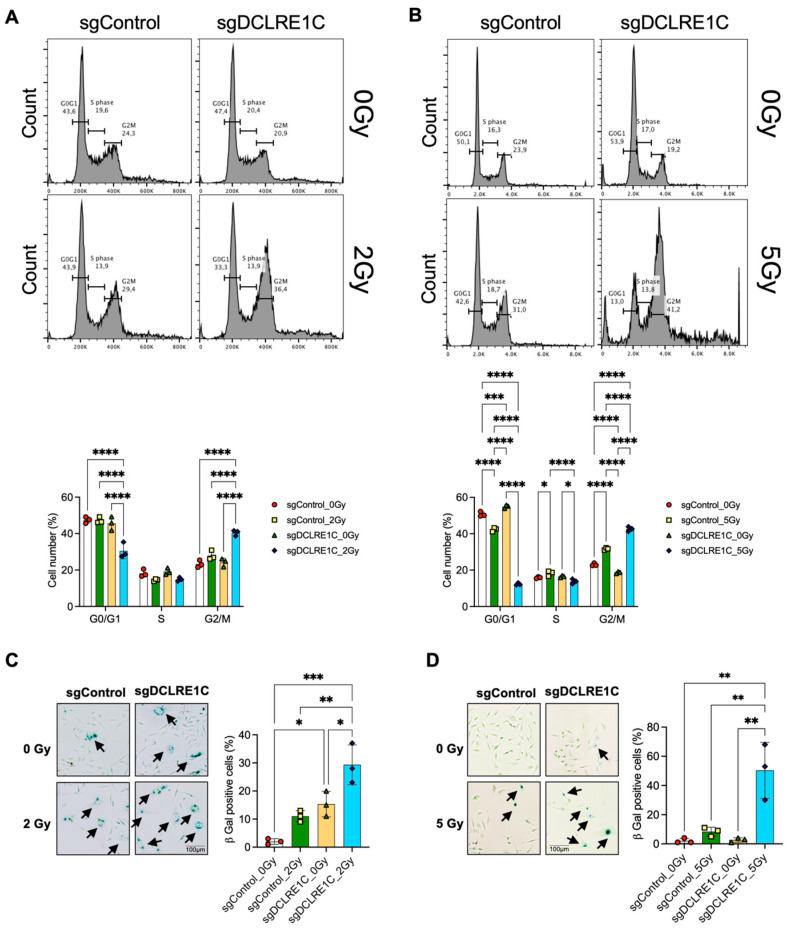
**Loss of Artemis expression enhances radiation response via the induction of G2/M arrest and cellular senescence**. Cell cycle analysis in TNBC cells was performed to assess the consequence of Artemis knockout, alone or in combination with RT. (**A**,**B**) Loss of Artemis combined with RT resulted in a significant increase in G2/M arrest compared to control, RT alone, and the Artemis knockout condition alone at 24 h in both (**A**) MDA-MB-231 and (**B**) SUM159 cells. A total of 10,000 events were captured. (**C**) Representative images (left) shows positive β-galactosidase staining in sgControl and sgDCLRE1C MDA-MB-231 cells. Bar graph (right) shows percentage of β-galactosidase positive cells in control, and Artemis knockout cells, alone or in combination with RT. (**D**) Representative images (left) shows positive β-galactosidase staining in sgControl and sgDCLRE1C SUM159 cells. Bar graph (right) shows percentage of β-galactosidase positive cells in control, and Artemis knockout cells, alone or in combination with RT. Arrows point at cells positive for β-galactosidase staining. * = <0.05, ** = <0.01, *** = <0.001, and **** = 0.0001. Scale = 100 µm.

Next, we chose to investigate the consequence of senescence signaling activation on the cell cycle in Artemis knockout cells following RT. Previous studies have demonstrated a bidirectional relationship between cell cycle arrest and senescence [66,67,68]. We found that Artemis knockout MDA-MB-231 cells (Supplementary Figure S3B), exposed to RT at 24 h had a significant increase in the percentage of cells in G2/M phase relative to control (1.9-fold; *p* value < 0.0001), RT alone (1.4-fold; *p* value < 0.0001), and Artemis knockout-alone (1.6-fold; *p* value < 0.0001) conditions (Figure 6A). Similarly, Artemis knockout SUM159 cells (Supplementary Figure S3C), exposed to 5 Gy RT showed a significant increase in the percentage of cells in G2/M phase relative to control (1.8-fold; *p* value < 0.0001), RT alone (1.3-fold; *p* value < 0.0001), and Artemis knockout alone (2.2-fold; *p* value < 0.0001) conditions (Figure 6B). Overall, our data suggested that Artemis knockout enhances RT response via the induction of G2/M arrest.

Our bulk RNA-seq revealed enrichment of the IL6/JAK/STAT3 signaling pathway, suggesting its potential role in the activation of cellular senescence and SASP. We observed that IL6 expression was significantly upregulated in Artemis knockout cells, both alone and in combination with RT (Figure 5D). This increase in IL6 mRNA correlated with higher IL6 protein levels in MDA-MB-231 Artemis knockout cells alone, which further increased in the presence of RT (Supplementary Figure S3D) relative to control. We also observed a similar increase in IL6 protein levels in SUM159 Artemis knockout cells following RT (Supplementary Figure S3F). Given that GSEA identified significant enrichment of the IL6/JAK/STAT3 signaling axis in both Artemis knockout, alone or in combination with RT, we assessed STAT3 phosphorylation. We found that STAT3 phosphorylation was significantly increased in Artemis knockout cells following RT relative to control in both MDA-MB-231 and SUM159 TNBC models (Supplementary Figure S3E,G).

Finally, to investigate whether the loss of Artemis-induced senescence in TNBC cells, we quantified cellular senescence using the β-galactosidase assay. In MDA-MB231 Artemis knockout cells, we observed a significant increase in the number of senescent cells as compared to control (*p* value = 0.02), which further increased in the presence of RT as compared to control (*p* value = 0.0003), RT alone (*p* value = 0.003) and Artemis knockout alone (*p* value = 0.01) (Figure 6C). Similarly, we observed a significant increase in the number of senescent cells in SUM159 Artemis knockout cells following RT as compared to control (*p* value = 0.0014), RT alone (*p* value = 0.0033), and Artemis knockout alone (*p* value = 0.0015) conditions (Figure 6D). Taken together, our data demonstrated that a combination of Artemis knockout and RT enhances anti-tumor effects by inducing G2/M arrest and activating cellular senescence.

## 4. Discussion

RT is a key treatment modality used for both the local and regional control of primary breast cancer and its metastases [4,5,7]. However, development of resistance to RT results in disease recurrence and poor treatment outcomes [69]. For an effective RT response, it is imperative to understand the molecular mechanisms underlying cellular responses to RT, which will help identify novel targets and biomarkers to enhance RT response in TNBC patients.

We used genome-wide CRISPR-Cas9 knockout screening in MDA-MB-231 cells and identified 172 potential radiosensitizing targets. Our screen revealed Artemis, a key component of the NHEJ pathway, as a top hit, whose depletion resulted in reduced cellular viability following RT. Previous studies have shown that loss of Artemis function [70,71,72,73,74] and, inherited mutations or polymorphic variants result in cellular radiosensitivity [75,76,77,78]. Our analysis of the breast cancer TCGA database revealed that *DCLRE1C* mRNA expression is significantly higher in TNBC, compared to normal breast tissue and other breast cancer subtypes (Figure 1E). Notably, TNBC patients with higher *DCLRE1C* mRNA expression had poor overall survival (Figure 1F). This was consistent with evidence highlighting the role of Artemis as a radioresistance gene in multiple cancers, including cervical, hepatobiliary, and colorectal cancers resulting in worse overall survival and poor prognosis [71,74,79,80,81]. These results emphasize the potential of Artemis as a biomarker and therapeutic target as a single agent or in combination with RT and potentially chemotherapy or immunotherapy [79,80,82].

Artemis deficiency has been reported to exhibit antiproliferative [70,71,74,80,81,83], pro-apoptotic [84], and anti-invasive [85] effects in various cancers. Our characterization of Artemis knockout TNBC models indicated that its depletion alone led to a significant decrease in proliferation and increase in tumor doubling times of TNBC cells, as well as improved survival of mice bearing tumor xenografts (Figure 3A,B). Additionally, our data also demonstrated that RT in Artemis-depleted models led to a significant improvement in the survival of mice bearing tumor xenografts compared to either Artemis depletion or RT alone (Figure 3A). These are novel findings, emphasizing the critical role of Artemis in TNBC pathogenesis and present us with an opportunity for targeting Artemis alone or in combination with RT or DNA-damaging chemotherapeutic agents to enhance anti-cancer treatment efficacy. Collectively, these findings suggest that Artemis holds potential as both a therapeutic target and a prognostic biomarker in TNBC.

To this end, we used clinically approved non-oncology drugs previously shown to inhibit Artemis activity in cell-free assays [27], since, to our knowledge, there are no clinically approved novel drugs to specifically inhibit Artemis. A previous study demonstrated that β-lactam antibiotics can be repurposed as a pro-senescent radiosensitizer in estrogen receptor-positive breast cancer cells [86]. The β-lactam antibiotics such as ceftriaxone [27], ampicillin [52], and auranofin [27], which displaces zinc (Zn) from the β-CASP domain have been shown to inhibit Artemis’ nuclease activity. Additionally, auranofin has been shown to sensitize colon cancer cells to RT in vivo and human colon cancer patient-derived organoids [87]. Our results showed that ceftriaxone and auranofin, but not ampicillin, significantly reduced colony formation, an effect further enhanced when combined with RT in both MDA-MB-231 and SUM159 cells (Figure 2C–F, Supplementary Figure S1D,E). However, both ceftriaxone [88,89] and auranofin [90,91] have been shown to exhibit additional mechanisms of anti-cancer effects which need to be further investigated. Our findings support further investigation into the potential repurposing of ceftriaxone and auranofin for TNBC treatment. These results also highlight the opportunity to develop novel structural analogs for enhancing radiotherapy efficacy in TNBC. While our in vitro findings support the potential to repurpose these drugs, we acknowledge that additional in vivo and clinical studies are needed to define achievable plasma concentrations, pharmacokinetics, and toxicity profiles, as well as to establish overall feasibility and safety of use in TNBC. In this study, we used a single-fraction ID50 RT dose to assess the role of Artemis as a therapeutic target. We recognize that this single ID50 dose regimen may not fully replicate clinically relevant fractionation in TNBC, such as multiple daily fractions or hypofractionation protocols. Future studies incorporating these clinical dosing schedules will be essential to determine the translational relevance and therapeutic potential of targeting Artemis in combination with RT.

Cellular senescence or apoptosis is often active in response to unrepaired DNA damage in cells [92]. Loss of Artemis has been shown to trigger apoptosis in osteosarcoma and cervical cancer cell lines [84]. However, in our study, DEG analysis revealed the perturbation of genes linked to cellular senescence (Figure 4D). Among these, *IL6*, a key component of SASP [93,94], was significantly upregulated in the absence of Artemis (Figure 4D). This upregulation was consistent in Artemis knockout cells exposed to RT (Figure 5D). Furthermore, the increase in *IL6* mRNA correlated with IL6 protein levels, which further increased in the presence of RT (Supplementary Figure S3D,F). Along with the enrichment of IL6/JAK/STAT3 signaling, our data suggested a possible IL6-mediated modulation of RT responses through the activation of cellular senescence. Interestingly, IL6/JAK/STAT3 signaling has been shown to have pro-tumorigenic roles in multiple malignancies [95]. This suggests that the IL6/JAK/STAT3 signaling axis may also activate a protective mechanism in Artemis knockout cells following RT. Therefore, the regulatory role of IL6/JAK/STAT3 signaling in regulating cellular senescence needs to be further investigated in Artemis knockout cells following RT. Interestingly, Artemis knockout cells exposed to RT exhibited even greater perturbation of senescence-related genes (Figure 5D). Consistent with this, we observed a significant increase in cellular senescence in Artemis knockout cells following RT (Figure 6C,D) assessed through the β-galactosidase assay. These findings suggest that loss of Artemis triggers cellular senescence, contributing to increased RT sensitivity.

Both unrepaired DNA damage [96] and cellular senescence [67] can trigger G2/M phase arrest and promote genomic instability [97]. Primary skin fibroblasts with mutant Artemis are associated with a defect in the G2 phase of cell cycle following RT [77]. Loss of Artemis has been shown to cause G1 arrest and subsequent apoptosis through stabilization of the p53 protein [84]. However, in our study, Artemis knockout cells exposed to RT exhibited significant G2/M phase accumulation 24 h post RT (Figure 6A,B). Taken together, our data suggest that the combination of Artemis knockout and RT results in the activation of cellular senescence and G2/M arrest, eventually leading to anti-cancer effects in TNBC cells. Interestingly, the overexpression of Artemis in skin fibroblasts derived from a radiosensitive breast cancer patient was associated with unrepaired DNA damage and enhanced apoptosis [98]. This suggests that the role of Artemis is time- and context-dependent and may differentially modulate RT responses in patients and requires further investigation.

## 5. Conclusions

For the first time to our knowledge, we show that Artemis plays an important role in TNBC proliferation, growth, and senescence. We also show that Artemis knockout facilitates RT responses through the activation of senescence pathways and cell cycle perturbations. Artemis knockout alone or in combination with RT leads to significant improvements in the survival of xenograft-bearing mice, and its mRNA expression correlates with patient outcomes in TNBC. Moreover, we investigated biological mechanisms of Artemis depletion with or without RT and found that Artemis knockout activates inflammatory and cellular senescence pathways and leads to G2/M arrest in response to RT. As such, Artemis appears to represent an important molecule in TNBC biology and bears significant potential as a treatment response biomarker and novel drug target. Its role in modulating cellular responses to DNA-damage and immune/inflammatory responses warrants further investigation into its utility as a therapeutic vulnerability in combination with RT and DNA-damaging chemotherapeutics and immunotherapies, respectively.

## Data Availability

The data generated in this study are available upon reasonable request.

## Supplementary Materials

The following supporting information can be downloaded at: https://www.mdpi.com/article/10.3390/cancers17203279/s1, Figures S1: (A) Schematic illustrates the experimental strategy for CRISPR-Cas9 genome wide screening. Schematic shows the CRISPR screen design and analysis for identification of potential radiosensitizing targets from the screen. (B,C) Western blotting shows the expression of Artemis in different TNBC knockout cell lines, (B) MDA-MB-231 and (C) SUM159, generated using individual sgRNA targeting the DCLRE1C gene. (D,E) Colony forming assay was performed in MDA-MB-231 and SUM159 TNBC cells exposed to different doses of ampicillin alone or in combination with ID50 doses of RT (2 Gy for MDA-MB-231 and 5 Gy for SUM159), **** =< 0.0001. (F) Schematic representation of the in vivo experiment to assess the rate of tumor growth of MDA-MB-231 cells under different treatment conditions; Figures S2: (A) Principal component analysis plot generated from RNA-Seq analysis shows clustering of sgControl and sgDCLRE1C cells exposed to either 0 Gy (NRT = No RT) or 2 Gy RT. (B) TCGA analysis shows transcript levels of cellular senescence-related genes in TNBC samples relative to luminal and HER2+ breast cancer subtypes and normal tissue samples. (C-D) RT-qPCR validation of cellular senescence related genes in (C) MDA-MB-231 and (D) SUM159 cells. * =< 0.05, ** =< 0.005, *** =< 0.001; Figures S3: (A) Dot plot shows overrepresented signaling pathways generated by comparing DEGs in Artemis knockout cells with and without RT. (B,C) Confirmation of Artemis expression in control and Artemis knockout lines through western blot analysis in (B) MDA-MB-231 and (C) SUM159 cells. (D) Western blot images (left) and densitometric analysis (right) show a significant increase in IL6 protein levels in MDA-MB-231 Artemis knockout cells, alone and in combination with RT. (E) Western blot images (left) and densitometric bar graph (right) show phosphorylated STAT3 (pSTAT3) to total STAT3 protein levels in MDA-MB-231 cells. (F) Western blot images (left) and densitometric analysis (right) show a significant increase in IL6 protein levels in SUM159 Artemis knockout cells, alone and in combination with RT. (G) Western blot images (left) and densitometric bar graph (right) show phosphorylated STAT3 (pSTAT3) to total STAT3 protein levels in SUM159 cells. * =< 0.05, ** =< 0.005, *** =< 0.001; Table S1A: The list of guide RNA sequences used to target Artemis (DCLRE1C) in this study; Table S1B: The list of antibodies used and conditions in this study; Table S2: The list of potential radiosensitizing targets identified in our CRISPR screen; Table S3: The list of enriched genes sets identified through GSEA under different comparisons: sgDCLRE1C_0Gy vs. sgControl_0Gy; sgDCLRE1C_2Gy vs. sgControl_0Gy and sgDCLRE1C_2Gy vs sgControl_2Gy; Table S4: The list of differentially expressed genes generated by comparing different experimental conditions: sgDCLRE1C_0Gy vs. sgControl_0Gy; sgDCLRE1C_2Gy vs. sgControl_0Gy and sgDCLRE1C_2Gy vs. sgControl_2Gy.
