# Supplementary figures and images for: Artemis (DCLRE1C) Acts as a Target to Enhance Radiotherapy Response in Triple-Negative Breast Cancer
Source: Cancers (Basel). 2025 Oct 10;17(20):3279. doi: 10.3390/cancers17203279 (PMC12564160; doi:10.3390/cancers17203279)

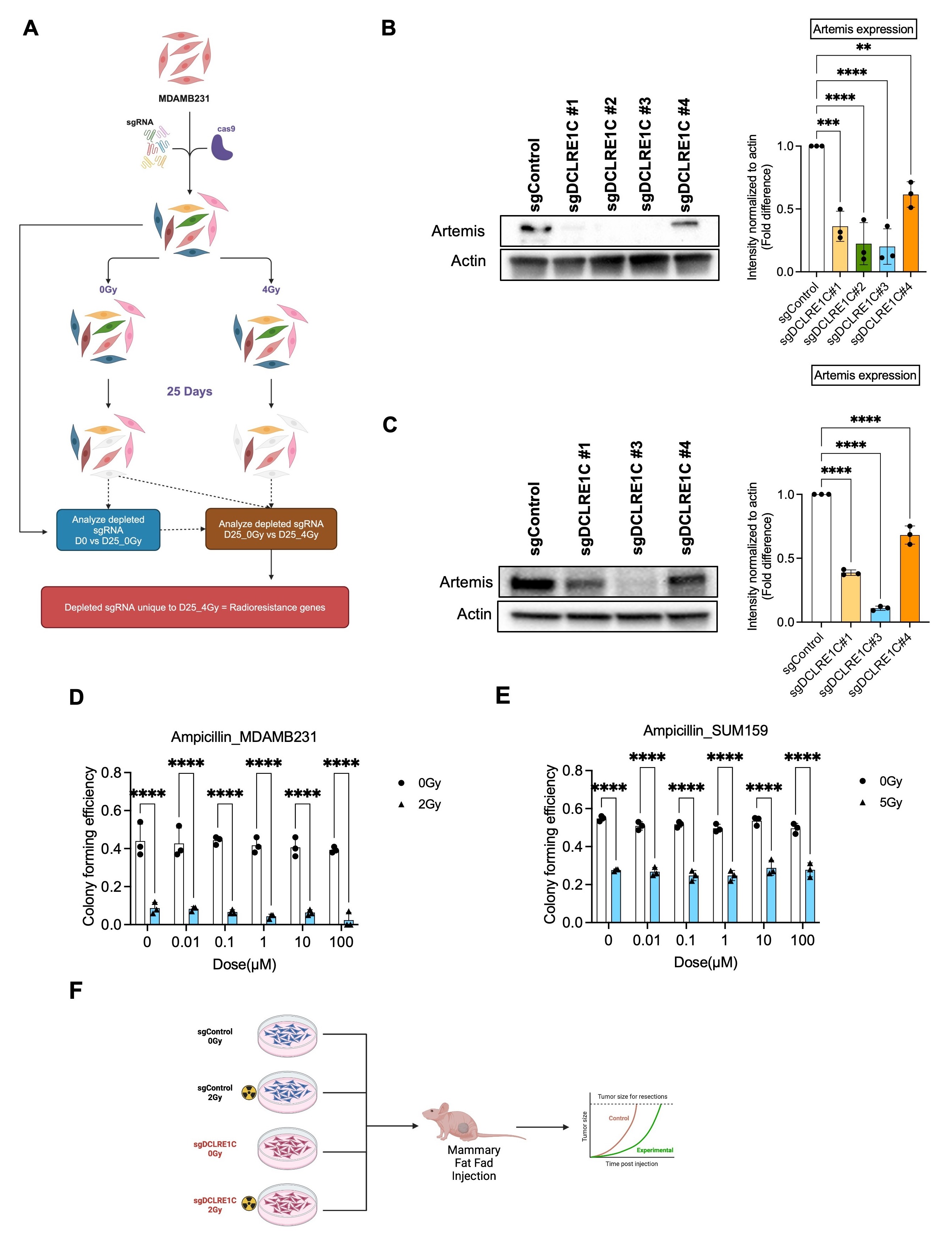

Supplement: Supplementary file 1 [file cancers-17-03279-s001.zip › Figure S1.jpg]

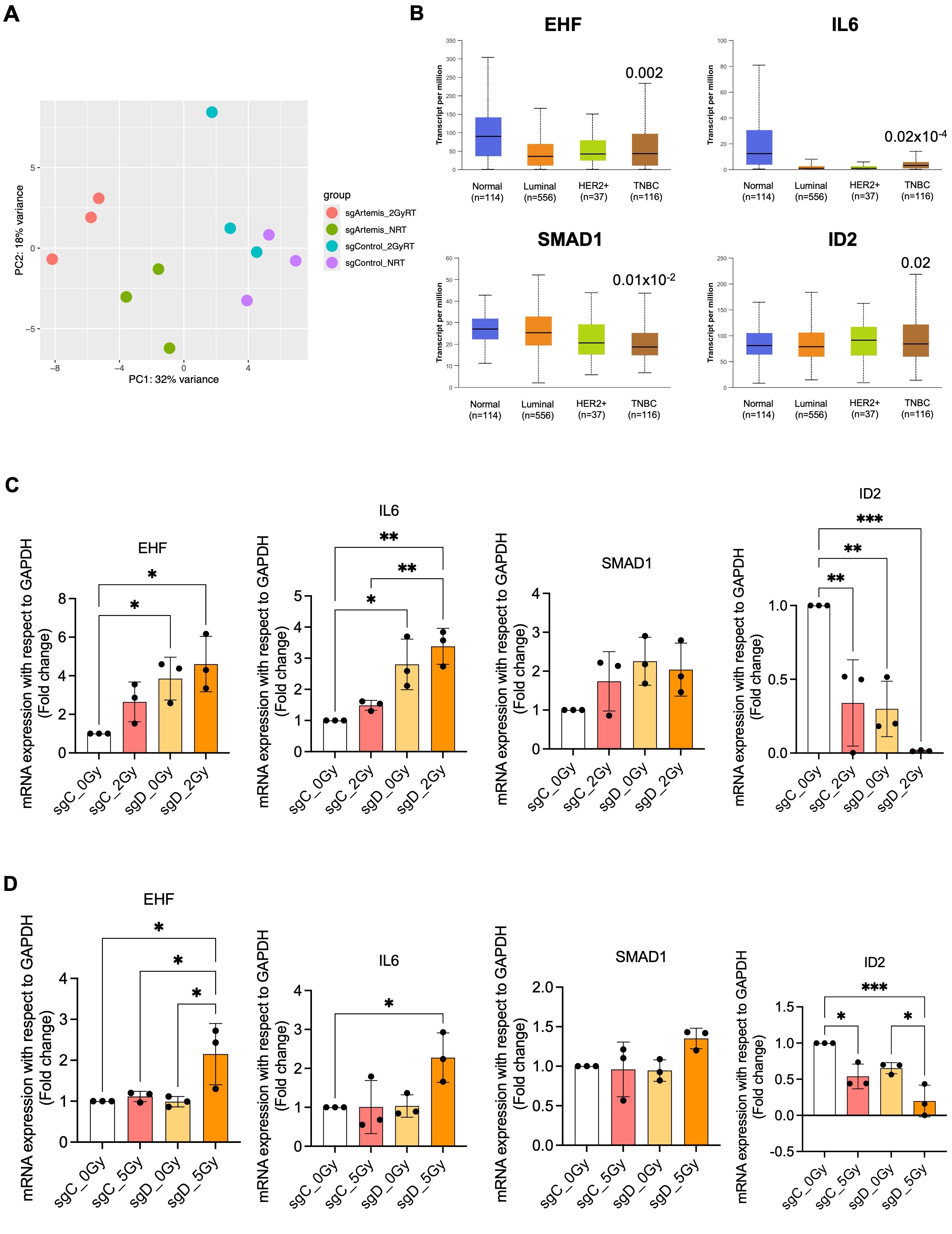

Supplement: Supplementary file 1 [file cancers-17-03279-s001.zip › Figure S2.jpg]

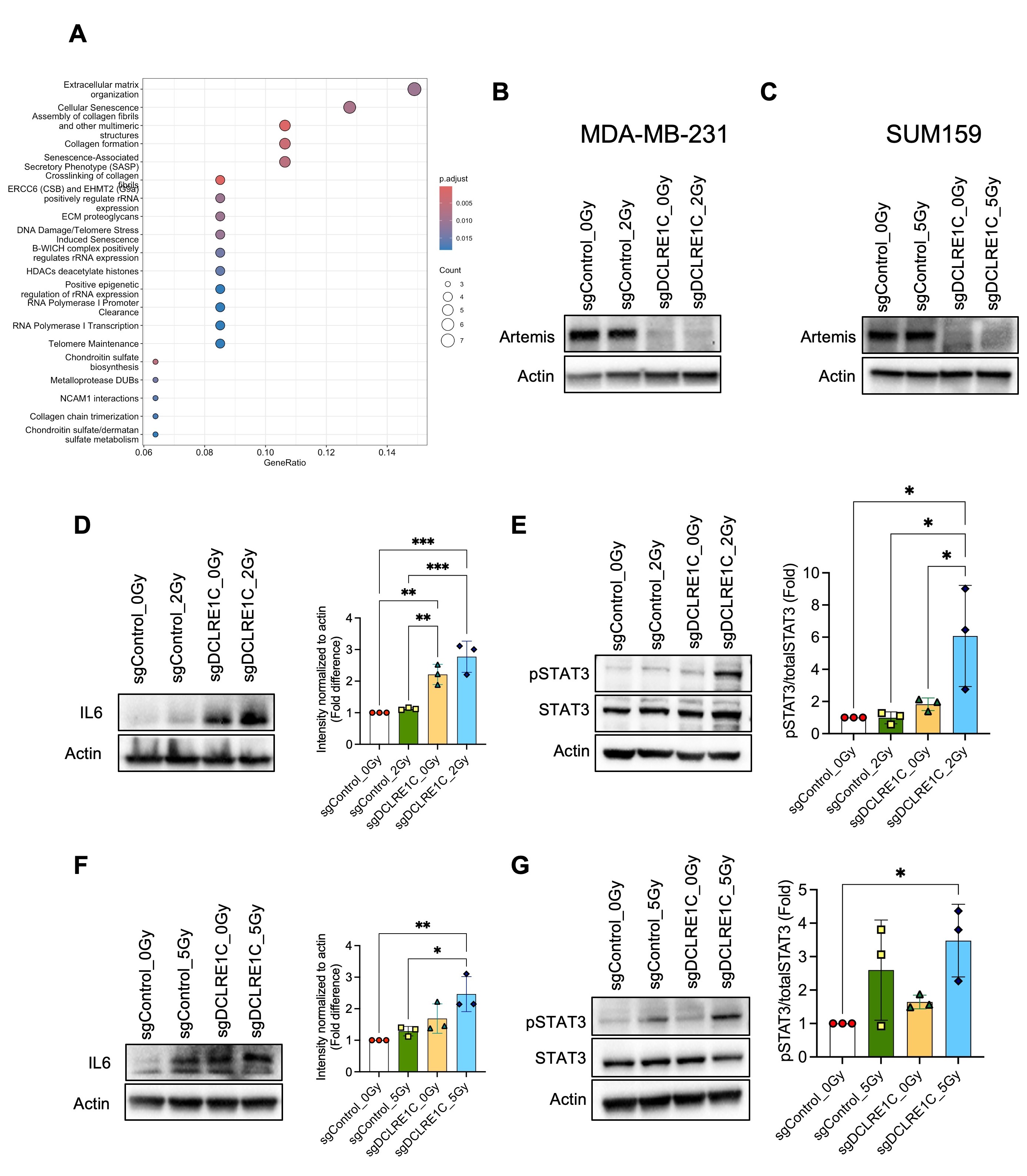

Supplement: Supplementary file 1 [file cancers-17-03279-s001.zip › Figure S3.jpg]
